# First Complete Genome Sequence of an Isolate of Tomato Mottle Mosaic Virus Infecting Plants of Solanum lycopersicum in South America

**DOI:** 10.1128/genomeA.00427-18

**Published:** 2018-05-10

**Authors:** Alice Nagai, Lígia M. L. Duarte, Alexandre L. R. Chaves, Maria A. V. Alexandre, Pedro L. Ramos-González, Camila Chabi-Jesus, Ricardo Harakava, Déborah Y. A. C. dos Santos

**Affiliations:** a Departamento de Botânica, Instituto de Biociências, Universidade de São Paulo, São Paulo-SP, Brazil; b Laboratório de Fitovirologia, Instituto Biológico, São Paulo-SP, Brazil; c Laboratório Bioquímica Fitopatológica, Instituto Biológico, São Paulo-SP, Brazil; d Escola Superior de Agricultura Luiz de Queiroz, Universidade de São Paulo, Piracicaba-SP, Brazil

## Abstract

The complete nucleotide sequence of an isolate of tomato mottle mosaic virus (ToMMV) was determined. The virus, originally isolated from symptomatic tomato plants found in a county near the city of São Paulo, Brazil, has a genome with 99% nucleotide sequence identity with ToMMV from Mexico, China, Spain, and the United States.

## GENOME ANNOUNCEMENT

Tomato mottle mosaic virus (ToMMV) is assigned to the genus Tobamovirus (family Virgaviridae) (1). This virus produces necrosis of the seedling leaves and mosaic and leaf distortion of mature tomato plants (2). ToMMV was first identified infecting tomatoes in Mexico in 2009 (2). Since then, it has been detected in the United States (3, 4), Spain (5), Israel (6), and China (7). A tomato plant showing severe mosaic, deformation, and tapering of the leaflet was collected at Capão Bonito municipality, in the state of São Paulo, Brazil, in 1992. Infection by a tobamovirus-like virus was suspected because of the detection of rod-shaped particles by transmission electron microscopy, reaction of sap extract with a tomato mosaic virus (ToMV) antiserum, biological indexing, and high nucleotide sequence identity of the coat protein gene (GenBank accession number KT222999) with that of the ToMMV isolate from Mexico (KF477193). In the present work, we describe the complete genome sequence of the Brazilian isolate of ToMMV (CpB1 isolate) and reveal its relationship with other tobamoviruses.

Sap extract from the infected tomato sample was used to infect Solanum pimpinellifolium plants. Upon symptom appearance, a virion-enriched solution, prepared following a protocol for purification of rod-shaped particles (8), was used to obtain a viral RNA solution by using TRIzol (Invitrogen, Gibco BRL, USA). A cDNA library was generated using the Illumina TruSeq Stranded mRNA sample preparation LS protocol, and sequencing was performed using Illumina HiSeq 2500 technology. Reads were assembled to obtain the final genome using CAP3 software version 2015-10-02. The sequence at the 5′ terminus of the genome was obtained by rapid amplification of cDNA ends (RACE) technology using an appropriate primer and following the manufacturer´s recommendation for the SMARTer RACE 5′/3′ kit (Clontech Laboratories, Inc., CA, USA). The complete sequence was compared with those from tobamoviruses retrieved from the GenBank database using the BLAST algorithm (9). Sequence alignments were carried out using Se-Al software version 1.0 alpha 1 (10).

The complete genome sequence of the CpB1 isolate is 6,383 nucleotides (nt) long and consists of four open reading frames (ORFs), annotated according to their identity with those typically found in known tobamoviruses. ORF1 (3,351 nt) encodes the methyltransferase and helicase, whereas a second ORF, which is 4,851 nt long and 5′ coterminal with ORF1, encodes the domain RNA-dependent RNA polymerase. ORF3 (807 nt) and ORF4 (480 nt) encode the movement protein and the coat protein, respectively. Pairwise alignment using the maximum composite likelihood model revealed that this isolate shared ~99% nucleotide sequence identity with the full genome sequences of ToMMV isolates from Mexico (GenBank accession number KF477193), Florida (KP202857), New York (KT810183), California (KX898034), China (KR824950), and Spain (KU594507).

Our results confirm the occurrence of ToMMV in Brazil since 1992. Moreover, they alert us to the putative misidentification of ToMMV in infected plants based on the use of ToMV antiserum. Consequently, both tomato producers and phytosanitary authorities should be aware about the possible underestimation of ToMMV incidence in Brazil. To the best of our knowledge, this work is the first to provide the complete genome sequence of a ToMMV isolate from South America.

### Accession number(s).

The complete genome of the Brazilian ToMMV isolate was deposited in GenBank under the accession number MH128145.
